# Combination Treatment of Withalongolide a Triacetate with Cisplatin Induces Apoptosis by Targeting Translational Initiation, Migration, and Epithelial to Mesenchymal Transition in Head and Neck Squamous Cell Carcinoma

**DOI:** 10.3390/nu14245398

**Published:** 2022-12-19

**Authors:** Chitra Subramanian, Katie K. Spielbauer, Robin Pearce, Kevin J. Kovatch, Mark E. Prince, Barbara N. Timmermann, Mark S. Cohen

**Affiliations:** 1 Departments of Surgery and Bioengineering, Carle Illinois College of Medicine, University of Illinois, Urbana-Champaign, IL 61820, USA; 2 Department of Otolaryngology-Head and Neck Cancer, Michigan Medicine, Ann Arbor, MI 48109, USA; 3 Department of Computational Medicine and Bioinformatics, Ann Arbor, MI 48109, USA; 4 Department of Otolaryngology, Danville, PA 17822, USA; 5 Department of Medicinal Chemistry, University of Kansas, Lawrence, KS 66045, USA

**Keywords:** heat shock protein 90, withalongolide, head and neck squamous cell carcinoma, cisplatin, and translation complex

## Abstract

Treatment regimens for head and neck squamous cell carcinoma (HNSCC) typically include cisplatin and radiotherapy and are limited by toxicities. We have identified naturally derived withalongolide A triacetate (WGA-TA) from *Physalis longifolia* as a lead compound for targeting HNSCC. We hypothesized that combining WGA-TA with cisplatin may allow for lower, less toxic cisplatin doses. HNSCC cell lines were treated with WGA-TA and cisplatin. After treatment with the drugs, the cell viability was determined by MTS assay. The combination index was calculated using CompuSyn. The expression of proteins involved in the targeting of translational initiation complex, epithelial to mesenchymal transition (EMT), and apoptosis were measured by western blot. Invasion and migration were measured using the Boyden-chamber assay. Treatment of MDA-1986 and UMSCC-22B cell lines with either WGA-TA or cisplatin alone for 72 h resulted in a dose dependent decrease in cell viability. Cisplatin in combination with WGA-TA resulted in significant synergistic cell death starting from 1.25 μM cisplatin. Combination treatment with WGA-TA resulted in lower cisplatin dosing while maintaining the downregulation of translational initiation complex proteins, the induction of apoptosis, and the blockade of migration, invasion, and EMT transition. These results suggest that combining a low concentration of cisplatin with WGA-TA may provide a safer, more effective therapeutic option for HNSCC that warrants translational validation.

## 1. Introduction

Head and neck squamous cell carcinoma (HNSCC) is the sixth most common cancer worldwide, with most of these cancers arising in the oral cavity and oropharynx [1]. The current standard of care involves a combination of surgery, chemotherapy, and radiation. Despite advancements in adjuvant treatments, survival in advanced HNSCC has remained relatively unchanged [2]. While there has been a rise in HPV-associated cancers of the oropharynx exhibiting improved clinical outcomes, the majority of advanced HPV negative HNSCCs continue to exhibit poor outcomes [1,2,3]. Current standard of care therapies, including cisplatin and cetuximab, demonstrate limited long-term efficacy due to the development of drug resistance and systemic toxicity at higher doses [4,5,6]. The toxicities of cisplatin are well characterized and traditionally include ototoxicity and renal toxicity. The limitations of surgery, radiation, and systemic chemotherapy in advanced disease have shifted focus to the development of safer and more durable alternative therapies.

Heat shock protein 90 (Hsp90) is a molecular chaperone which aids several “client” proteins in conformational folding, allowing for the phosphorylation and activation of these proteins and their pathways, many of which are hallmarks of carcinogenesis [7,8,9]. Thus, the inhibition of the hetero chaperone complex function has broad therapeutic potential in cancers due to the ability to suppress multiple oncogenic pathways simultaneously, including cell growth and proliferation pathways commonly amplified in HNSCC. The inhibition of Hsp90 has been proposed and studied clinically in the treatment of several cancers and has shown excellent anti-tumoral response in HNSCC and other cancers [10]. However, these Hsp90 inhibitors have demonstrated dose-limiting toxicities as a monotherapy in phase II studies limited primarily due to hepatotoxicity. It has been postulated that limited dose escalation to the maximal tolerated dose (MTD) observed in clinical trials may be related to the mechanism by which inhibition of Hsp90 at the amino-terminal binding site leads to the displacement of heat shock transcription factor 1 (HSF-1). HSF-1 release induces the “heat shock response” with the upregulation of pro survival effects mediated through Hsp70, thus requiring additional Hsp90 dose inhibition to maintain cancer growth control until MTD and dose limiting toxicities (DLTs) are reached [9,11]. Known limitations to single drug therapies as outlined above suggest that combination therapies may be necessary to identify clinically beneficial treatment regimens. Indeed, multidrug therapy has the potential to inhibit multiple key regulatory/dysregulated pathways simultaneously and to combat the development of resistance [12]. Further, synergistic effects in combination treatment may allow lower relative doses of each respective drug, leading to an improved systemic toxicity profile with potential for more durable treatment effects, as resistance to multidrug regimens have been shown to occur less frequently than with monotherapies.

Withanolides are novel naturally derived compounds present in the Solanaceae family of plants. The well-known withanolide, withaferin A, has been used as a safe nutraceutical therapeutic option in Indian Ayurvedic medicine for centuries [13]. Through structure-activity-relationship studies, we have identified and purified a naturally derived inhibitor of the hetero chaperone complex, withalongolide A triacetate (WGA-TA), from *Physalis longifolia*, as a hit compound for targeting several cancers including HNSCC [14]. Withanolides, including WGA-TA, are plant derived nutraceutical compounds that are widely used in Ayurvedic medicine for a variety of diseases [13]. Withalongolide A triacetate (WGA-TA) is a safe biosynthetic withanolide which inhibits Hsp90 client proteins by disruption of the Hsp90 hetero chaperone complex through disrupting the co-chaperone cdc37 from Hsp90, leading to the induction of apoptosis and the inhibition of several regulatory pathways including PI3K/Akt/mTOR, which has been implicated in cisplatin resistance and HNSCC progression [15]. mTOR, in addition to coordinating upstream regulators, also regulates translation via 4E-BPs and S6Ks, which in turn modulates the downstream regulators like e1F4B [16]. Therefore, we hypothesized that combining the novel compound WGA-TA with cisplatin would generate synergistic combined effects against HNSCC cancer growth, invasion, and metastatic potential at lower, less toxic doses of cisplatin.

In the present study, we evaluate treatment responses including cell viability, cellular proliferation, epithelial to mesenchymal transition (EMT), apoptosis, cellular pathways classically implicated in carcinogenesis and cisplatin resistance (i.e., PI3K/Akt/mTOR pathway), and cancer stem cell properties of WGA-TA and cisplatin combination treatment as a viable safe treatment option for HNSCC.

## 2. Materials and Methods

### 2.1. Cell Viability Assay

An MTS assay was used to determine the cell viability of the treated cells. Two HNSCC cell lines, MDA-1986 (oral cavity, generous gift from Dr. Jeffrey Myers, Houston, TX, USA, CVCL_6982 Expasy) and UMSCC-22B (larynx, generous gift from Dr. Thomas Carey, Ann Arbor, MI, USA), were cultured and seeded in 96-well plates at 1500 cells/well and 2500 cells/well, respectively. At 24 h after plating, cells were treated with a drug containing media at various concentrations and combinations of cisplatin and WGA-TA. After an additional 72 h incubation period, cell viability was assessed using a CellTiter96 Aqueous MTS assay with an absorbance measured at 490 nm on a Biotek Synergy 2 plate reader (BioTek, Winooski, VT, USA). IC50 values and combination indices were calculated for cisplatin and WGA-TA in both cell lines using GraphPad Prism 9 (GraphPad Software, Inc., La Jolla, CA, USA) and CompuSyn (ComboSyn Inc., Paramus, NJ, USA), respectively.

### 2.2. Western Blot Analysis

The efficacy of combination treatment was further characterized through western blot analysis of the MDA-1986 cell line. Cells reaching 40–60% confluence were treated for 24 h with cisplatin (1.25 or 2.5 μM), WGA-TA (0.125, 0.25, or 0.5 μM), or with both cisplatin and WGA-TA at each of the given concentrations. After treatment, cells were collected, pelleted, resuspended in lysis buffer, and sonicated. The protein concentrations were determined and standardized using a BSA protein assay. Approximately 20 μg of proteins was loaded on to the gel and the samples were separated by sodium dodecyl sulfate-polyacrylamide gel electrophoresis (SDS-PAGE). Samples were then transferred to nitrocellulose membranes and were blotted with primary antibody at 4 °C overnight. The following day, the membranes were blotted with secondary antibody for 1 h at room temperature, treated with Super Signal chemiluminescent reagent West PICO or FEMTO (Thermo Fischer Scientific, Waltham, MA, USA) for 1–5 min, and were captured on Kodak X-Ray film (Eastman Kodak, Rochester, NY, USA) or by using a BioRad chemidoc imaging system. ImageJ software (National Institutes of Health, Bethesda, MD, USA) was used to analyze the density of Western blot bands relative to the control band.

### 2.3. Boyden Chamber Assay for Migration and Invasion

Approximately 100,000 MDA-1986 cells in serum free medium were plated on the top insert of the Boyden Chamber insert. The cells were treated with either 1.25 or 2.5 μM cisplatin alone or in combination with 0.25 or 0.50 μM TA-WGA for 24 h. Post treatment, the cells were fixed with 2% paraformaldehyde, stained with 1% crystal violet in 20% methanol, washed and imaged using an EVOS microscope (Thermo Fisher Scientific, Waltham, MA, USA). Control inserts were used for migration and Matrigel coated inserts were used for invasion.

### 2.4. Statistical Analysis

All cell viability data was obtained in triplicate and were presented as mean ± standard deviation. Statistical differences between different treatments were calculated using a student’s *t*-test. Western blot quantification was done using ImageJ software (ImageJ, NIH).

## 3. Results

### 3.1. Determination of IC50 Values for Single Drug Treatment

**Figure 1:**
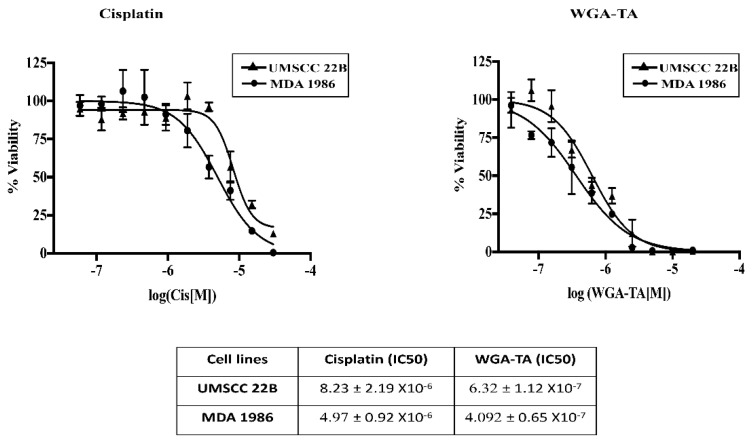
MDA-1986 and UMSCC-22B cells were treated with varying concentrations of either cisplatin alone or WGA-TA alone and the cell viability after 72 h was measured via MTS assay. The IC50 values (μM) shown below were calculated using GraphPad Prism.

The first step in our experimental design was to assess the viability of HNSCC cells following treatment with either cisplatin or WGA-TA monotherapy. MDA-1986 and UMSCC-22B cells were treated with serial dilutions of each drug (20 μM to 0.0195 μM). Cell viability was used to determine IC50 values using GraphPad Prism software as described above. A dose dependent treatment response was observed for each drug in both cell lines, indicated by a progressive decrease in cell viability. For both cell lines, IC50 values for WGA-TA were in the lower nanomolar range, whereas it was in the micromolar range for cisplatin. IC50 values for WGA-TA were up to 10-fold lower than that of cisplatin for UMSCC-22B and MDA-1986 cells (Figure 1).

### 3.2. Synergistic Effect of Combination Treatment

After determining IC50 values for each single drug treatment, combination treatment with WGA-TA and cisplatin was assessed. By calculating combination indices (CI) in response to dual-therapy treatment using the Chou-Talalay method [17], the synergistic effect between the drugs was determined. Using this method, synergistic, additive, and antagonist effects are indicated by CI < 1.0, CI = 1.0, and CI > 1.0, respectively. A synergistic effect (CI < 1) was observed in both cell lines across a range of concentrations after 72 h treatment with cisplatin and WGA-TA (Table 1A). Combination treatment resulted in a significant decrease in cell viability when compared with single drug treatment (*p* < 0.01 for combination vs. either drug alone). To determine suitable concentrations of a drug for subsequent western blot analysis, combination indices for 24 h treatment of MDA-1986 with WGA-TA and cisplatin were calculated. At this shorter treatment duration, we redemonstrated that WGA-TA and cisplatin act synergistically when several different concentrations of WGA-TA are combined with 1.25 and 2.5 μM cisplatin (Table 1A,B).

### 3.3. Effect of WGA-TA and Combination Treatment on the mTOR Pathway and Its Substrates 

**Figure 2:**
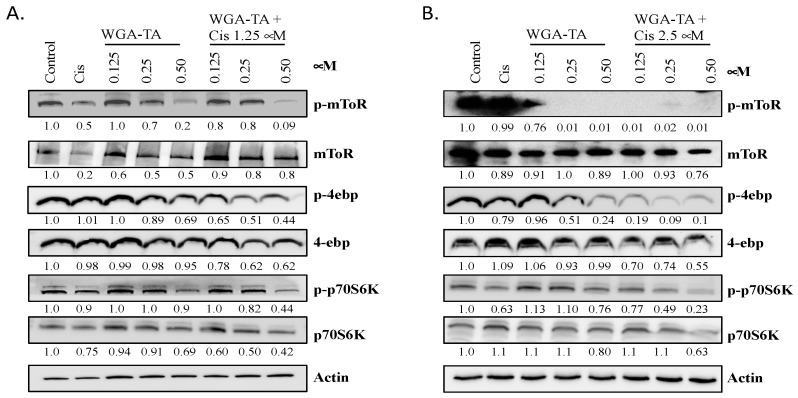
(**A**,**B**). Western blot showing treatment effect on the mTOR pathway and downstream substrates. MDA1986 cells were treated with varying concentrations of WGA-TA alone or in combination with 1.25 (**A**) or 2.5 (**B**) μM cisplatin as well as cisplatin alone for 24 h. Solvent treated cells were used as control. Cells were collected, lysed, and immunoblotted for mTOR pathway proteins.

Next, we examined how WGA-TA monotherapy and combination treatment modulated the mechanistic target of rapamycin (mTOR) and its protein substrates, specifically p70s6k and 4E-BP1 **(**Figure 2A,B). mTOR protein levels remained relatively consistent for each treatment; however, phosphorylated-mTOR (p-mTOR) levels declined when treated with either WGA-TA alone or the combination treatment. Treatment with 0.25 and 0.5 μM WGA-TA resulted in nearly full blockage of expression and combination treatment at the 2.5 μM cisplatin fully blocked expression of p-mTOR. The efficacy of combination treatment is most apparent at 0.125 μM WGA-TA, where combining cisplatin and WGA-TA resulted in a significantly greater reduction in p-mTOR levels than treatment with WGA-TA alone (*p* < 0.05). While p-mTOR levels decreased with treatment, 4E-BP1, p70s6k and phospho-p70s6k levels remained relatively constant. In contrast, p-4E-BP1 levels were significantly reduced. Combination treatment reduced p-4E-BP1 expression by 45–67% and 56–71% for WGA-TA plus 1.25 and 2.5 μM cisplatin, respectively. Treatment with 0.5 μM WGA-TA also significantly reduced expression levels. A clear synergistic effect in diminishing p-4E-BP1 expression can be seen for treatment with 0.125 and 0.25 μM WGA-TA alone and for their corresponding combinations with cisplatin.

### 3.4. Effect of WGA-TA and Combination Treatment on Translation Initiation

**Figure 3:**
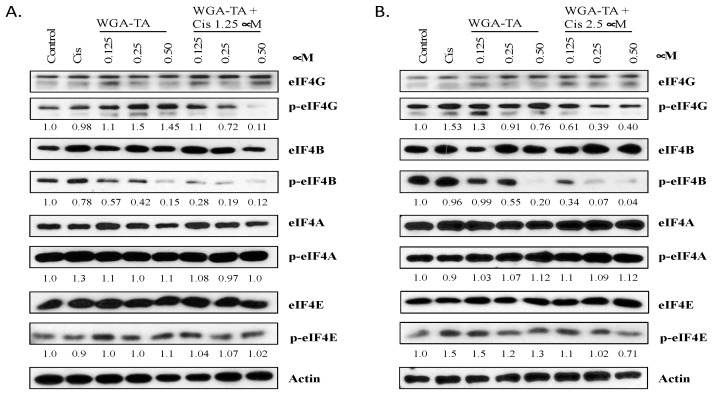
(**A**,**B**). Western blot showing the treatment effect on translation initiation. MDA-1986 cells were treated with either 1.25 (**A**) or 2.5 (**B**) µM cisplatin either alone or in combination with either 0.125 or 0.25 or 0.50 µM TA-WGA for 24 h. Solvent treated samples served as control. Post-treatment cells were collected, and equal amounts of proteins (20 µg) were loaded on SDS-PAGE gel and transferred onto a nitro cellulose membrane. The membranes were incubated with primary antibodies of translation complex proteins. The membranes were then treated with appropriate secondary antibodies and the blots were then developed using ECL.

As levels of the important translation regulation protein p-4E-BP1 diminished with treatment, we next studied the effect of WGA-TA and combination treatment on translation (Figure 3A,B).

To investigate the effect that treatment has on translation initiation, a western blot analysis was carried out on critical eukaryotic translation initiation complex proteins. Our results indicate that neither WGA-TA alone nor WGA-TA in combination with cisplatin modulate levels of eIF4G, eIF4B, eIF4A, eIF4A1, eIF4E, or p-eIF4E at the concentrations used in this study. Nonetheless, p-eIF4G and p-eIF4B expression levels were significantly attenuated in combination treatments compared to untreated or single agent treatment (*p* < 0.01). While treatment with cisplatin or WGA-TA alone had no effect on p-eIF4G levels, combination treatment resulted in a 42–44% decrease in p-eIF4G (*p* < 0.01), thereby decreasing translation. In addition, treatment with 0.125–0.5 μM WGA-TA decreased p-eIF4B expression by 23–90% in a dose-dependent manner (*p* < 0.01 for 0.5 μM WGA-TA vs. untreated), whereas combination with 1.25 and 2.5 μM cisplatin resulted in a 54–92% and a 70–96% reduction in expression, respectively. Even at low concentrations of cisplatin, a clear combination effect can be seen. The synergistic effect of combination is most notable for treatment with 0.25 μM WGA-TA. An amount of 0.25 μM WGA-TA alone attenuated expression of p-eIF4B by 35%, whereas combination with 1.25 and 2.5 μM cisplatin reduced expression by 83% and 93% (*p* < 0.01 vs. drug alone at the same concentration), respectively. Treatment with cisplatin alone did not inhibit the expression of p-eIF4B.

### 3.5. Effect of WGA-TA and Combination Treatment on the EMT, Apoptosis, Migration and Invasion

**Figure 4:**
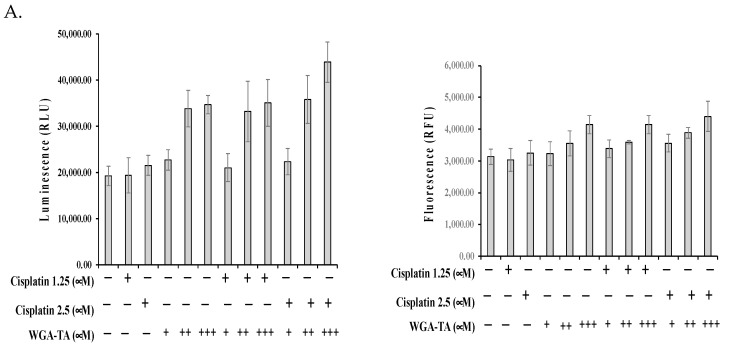

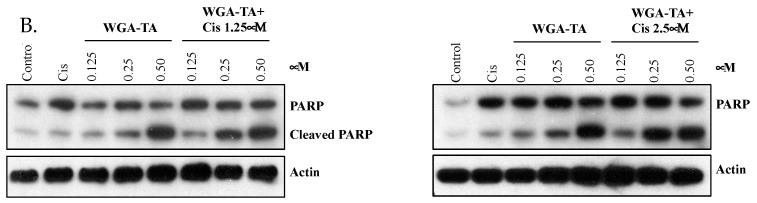
**A** (**Top**)**.** MDA1986 cells were treated either alone or in combination for 24 h. The induction of apoptosis and necrosis was detected using a RealTime-Glo annexin V apoptosis necrosis assay. The increase in luminescence induction of apoptosis and changes in fluorescence indicates the induction of secondary necrosis. **B** (**Bottom**). Western blot showing treatment effect on protein markers for the induction of apoptosis. MDA-1986 cells were treated with either cisplatin or WGA-TA alone or in combination for 24 h. Post-treatment cells were immunoblotted for PARP. Actin was used as a loading control.

**Figure 5:**
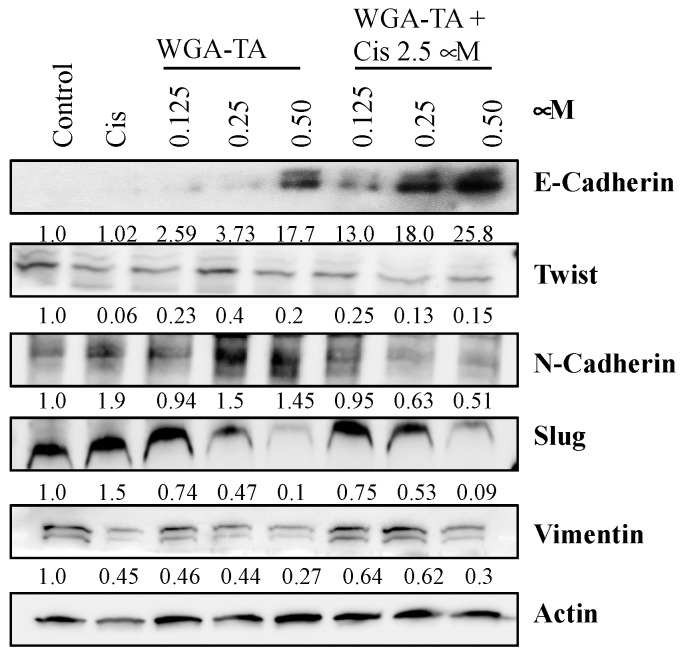
Immunoblot analysis of MDA-1986 cells treated with either 1.25 or 2.5 μM cisplatin alone or in combination with 0.25 or 0.50 μM WGA-TA for 24 h.

**Figure 6:**
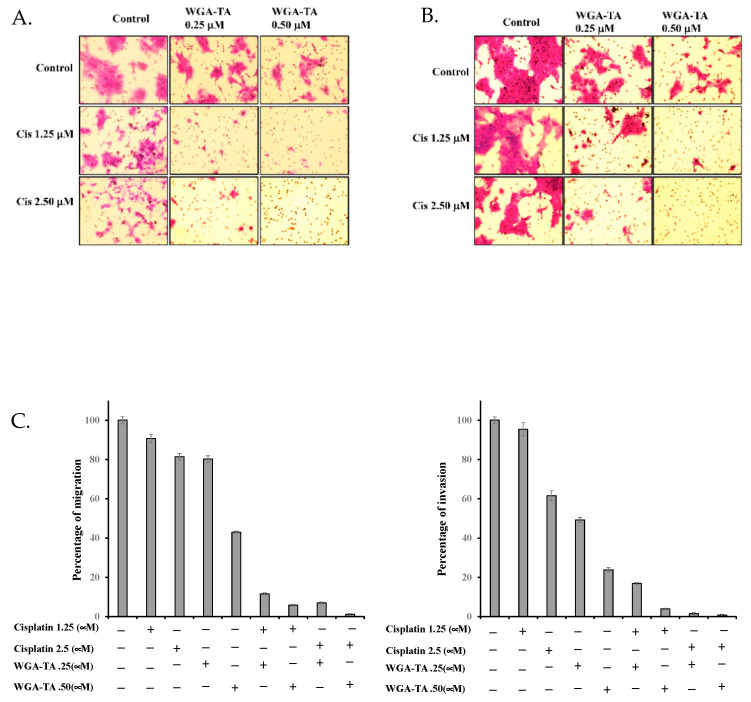
(**A**,**B**) The Boyden chamber assay utilized approximately 100,000 MDA-1986 cells treated with either 1.25 or 2.5 μM cisplatin alone or in combination with 0.25 or 0.50 μM WGA-TA for 24 h. Control inserts were used for migration (**A Left**) and Matrigel coated inserts were used for invasion (**B Right**). Post-treatment, the cells were fixed with 2% paraformaldehyde, stained with 1% crystal violet in 20% methanol, washed and imaged using a light microscope. Quantification for the migration and invasion are given below (**C**).

Finally, we investigated the degree to which WGA-TA and combination treatment induces cell death (Figure 4A,B) and alters expression levels for proteins involved in the epithelial-mesenchymal transition (EMT) (Figure 5), and migration and invasion (Figure 6). First, the mechanism of cell death was assessed using a RealTime-Glo apoptosis necrosis assay (Promega, WI, USA). The combination treatment of MDA1986 cells with cisplatin and WGA-TA for 24 h resulted in an increase in luminescence (phosphatidylserine Annexin V binding) and fluorescence (membrane integrity), suggesting the induction of apoptosis and secondary necrosis (Figure 4A). The induction of apoptosis was further studied by assessing PARP cleavage (Figure 4B). Both treatment with WGA-TA and combination treatment increased PARP cleavage by more than 10-fold (*p* < 0.01). Furthermore, combination treatment with 1.25 and 2.5 μM cisplatin and 0.25 μM WGA-TA resulted in approximately 2.5-fold more PARP cleavage than treatment with 0.25 μM WGA-TA alone (*p* < 0.01). 

Next, EMT transition was assessed by immunoblot analysis of EMT marker proteins. E-cadherin expression was nearly absent in the control, cisplatin, and 0.125–0.25 μM WGA-TA treatment groups (Figure 5). However, levels drastically increased with combination treatment, especially at 2.5 μM cisplatin (1.7–5.8-fold with combination treatment compared to single treatment, *p* < 0.01). In addition to e-cadherin, we also observed the down regulation of mesenchymal markers twist1, slug, vimentin and N-cadherin. These findings suggest the significant inhibition of EMT in response to combination treatment compared to each drug alone. 

Finally, the migration and invasion of MDA1986 cells after treatment with WGA-TA, cisplatin alone or in combination was assessed by Boyden chamber assay. The results shown in Figure 6A–C indicated a dose-dependent decrease in migration and invasion after treatment with either cisplatin or WGA-TA alone, whereas the combination treatment resulted in greater prevention of migration and invasion of cells even at 1.25 μM and 0.25 μM WGA-TA.

## 4. Discussion

There is a pressing need for more effective, durable, and less toxic treatment options for HNSCC, as survival rates for patients with advanced or recurrent cancers remain dismal [3]. Additionally, treatment with standard of care chemotherapeutic agents such as cisplatin is often rendered ineffective by the development of drug resistance, leading to persistent or recurrent disease in patients over time. The molecular chaperone function of the Hsp90 hetero chaperone complex interacting with many client proteins (especially kinases that accumulate preferentially in tumor cells) makes its inhibition a promising anti-cancer therapy [18]. Given the benefits of multimodal therapies in HNSCC, it is unlikely that single drug treatment with an Hsp90/hetero chaperone complex inhibitor will replace current treatment strategies combining chemotherapy with radiation; however, it may serve as a unique and important addition to current regimens to enhance efficacy and lower cisplatin toxicity through its synergistic effects with cisplatin at lower (less toxic) doses. Targeting multiple dysregulated oncogenic pathways simultaneously through combination therapies utilizing an Hsp90/hetero chaperone complex inhibitor like WGA-TA, may also be effective in overcoming cisplatin resistance and provide a more durable treatment response, as we have demonstrated that WGA-TA targets key resistance pathways in HNSCC, namely MAPK and PI3K/Akt/mTOR. Thus, the combination treatment presented here that involves WGA-TA and cisplatin represents an attractive strategy for providing effective, less toxic treatment in aggressive HNSCCs.

In this study, we describe a treatment strategy using WGA-TA alone and in combination with cisplatin. WGA-TA is a novel biosynthetic withanolide that blocks the Hsp90 hetero chaperone complex function by disrupting the binding of the co-chaperone, cdc37, to Hsp90. We demonstrated that this novel hetero chaperone complex inhibitor can create a synergistic treatment response when combined with standard of care cisplatin in aggressive HNSCC cell lines such as the MDA-1986. Combination indices using WGA-TA with cisplatin showed a synergistic response at many concentrations after both 72 h and 24 h treatment (all CIs < 0.9, several <0.4 for MDA-1986) including a markedly synergistic response in UMSCC-22B cells with several CIs < 0.1 (Table 1). The 24 h synergistic responses confirmed that shorter treatment conditions were appropriate for protein analysis by western blot analysis. This combination of WGA-TA and cisplatin was used to study HNSCC cell response in the aggressive oral cavity MDA-1986 cell line.

The mammalian target of rapamycin (mTOR) is a protein kinase that plays a crucial role in cell growth and homeostasis, which is often dysregulated in cancer cells, and upregulated in cisplatin resistant and aggressive HNSCCs. A western blot analysis indicated that treatment with WGA-TA alone and in combination with cisplatin therapy significantly reduced the expression of p-mTOR and p-4E-BP1 (one of its important downstream substrates). In its unphosphorylated state, 4E-BP1 binds eIF4E, a translation initiation factor. When eIF4E is bound by 4E-BP1, it is unable to promote translation, thus decreasing translation levels. However, when 4E-BP1 is phosphorylated, it releases eIF4E and translation levels increase. We observed that treatment with 0.125–0.5 μM WGA-TA significantly reduced p-mTOR levels, with 0.25 and 0.5 μM WGA-TA almost completely knocking down expression. Moreover, combination treatment at all three concentrations of WGA-TA with 2.5 μM cisplatin fully blocked the expression of p-mTOR. The additive effect of combination treatment can be most clearly seen at 0.125 μM WGA-TA, where combining cisplatin and WGA-TA resulted in a much greater reduction in p-mTOR levels than treatment with WGA-TA alone.

Proteomic alterations during mRNA translation by cancer cells plays an important role in cancer development, therapy resistance, and metastasis [19]. In prostate cancer, esophageal cancer, lymphoma and acute myeloid leukemia, the over expression of e1FE is known to promote therapy resistance and cancer progression [20,21,22,23]. The aberrant expression of e1F4G expression has also been linked to aggressive behavior in both breast and lung cancers [24,25]. During the critical rate-limiting step of translation initiation, cap-dependent protein eIF4E recruits eIF4G, which serves as a scaffold to which eIF4E and eIF4A/eIF4A1 bind during the eukaryotic translation initiation factor 4F (eIF4F) complex formation [26]. Oncogenic signaling pathways are known to augment the initiation of translation through altered expression and phosphorylation of translation initiation complex factors [27]. Therefore, in addition to evaluating upstream mTOR and 4E-BP1, we also evaluated the targeting of downstream translation machinery elements after combination treatment. Downstream of mTOR and 4E-BP1, the combination treatment of WGA-TA and cisplatin significantly attenuated the expression levels of several key eukaryotic translation initiation complex proteins, namely p-eIF4G and p-eIF4B. While treatment with cisplatin and WGA-TA had no effect on p-eIF4G levels, combination treatment resulted in a 42–44% decrease in p-eIF4G, thereby decreasing translation. The other translation initiation complex protein that was diminished by treatment was p-eIF4B. eIF4B acts alongside eIF4F to promote translation initiation. Like eIF4G, phosphorylation of eIF4B results in an increase in translation [28]. The phosphorylation of eIF4B was reduced 23–90% by treatment with 0.125–0.5 μM WGA-TA alone and by 54–92% and 70–96% for combination treatment with 1.25 and 2.5 μM cisplatin, respectively. A synergistic effect was observed for combination treatment, especially at 0.25 μM WGA-TA, where expression of p-eIF4B was significantly attenuated by 83–93% when combined with 1.25 or 2.5 μM cisplatin compared to 35% attenuation with WGA-TA alone. The down regulation of p-e1F4B is known to be involved in the regulation of several pro survival and proliferation mRNAs. This suggests that combination treatment of WGA-TA and cisplatin may be effective in controlling growth and inducing apoptosis through the synergistic inhibition of the mTOR pathway and its downstream substrates.

Next, we investigated protein markers of epithelial to mesenchymal transition (EMT), a process indicative of a greater propensity of cancer cells toward invasion and metastases. A primary feature of the EMT is the decreased expression of epithelial marker E-cadherin with the subsequent increased expression of the mesenchymal marker Vimentin. E-cadherin influences calcium-dependent cell-to-cell adhesion and inhibits the growth/metastasis of epithelial cancers, while Vimentin is a mesenchymal intermediate filament that helps coordinate various signaling pathways and has been shown to be elevated in metastatic tumors [29]. EMT quiescence was seen to a much greater degree with combination treatment relative to WGA-TA or cisplatin alone. This was especially true for combination treatment with 2.5 μM cisplatin, where combination with as low as 0.125 μM WGA-TA partially restored E-cadherin expression, and combination with 0.25 and 0.5 μM WGA-TA significantly increased E-cadherin levels. These results suggest that a WGA-TA and cisplatin combination treatment may be effective in blocking the invasion and metastatic potential in aggressive HNSCCs.

Finally, we studied the effect that WGA-TA treatment in combination with cisplatin had on apoptosis and cancer cell migration and invasion. To accomplish this, we screened for PARP and cleaved PARP levels on western blot. PARP is a protein involved in the repair of DNA breaks in response to environmental stress, and thus PARP cleavage indicates apoptosis through facilitation of irreversible cellular disassembly [30]. While higher concentrations of WGA-TA alone increased PARP cleavage significantly compared to controls or cisplatin alone, we observed an even more dramatic effect with a combination treatment at much lower doses of each drug, resulting in approximately 2.5-fold more PARP cleavage than treatment with WGA-TA monotherapy, suggesting that combination treatment (at safer doses) can effectively induce HNSCC cell death via apoptosis. The assessment of migration and invasion by Boyden chamber assay confirmed that combination treatment was highly effective in preventing the migration and invasion of HNSCC cells.

## 5. Conclusions

Inhibitors of the Hsp90 hetero chaperone complex, such as WGA-TA, are a promising treatment option for HNSCC. Hsp90’s clients include a diverse group of proteins that play a role in the development and proliferation pathways of numerous cancers. In this study, we demonstrated that the Hsp90 inhibitor, WGA-TA synergistically promotes apoptosis and inhibits HNSCC cell growth, migration, and invasion in combination with cisplatin in vitro. Specifically, these combination therapies effectively target a variety of pathways that are upregulated with cisplatin resistance, such as MAPK and the PI3K/Akt/mTOR pathway and its substrates. Specifically, combination treatment significantly upregulates translation initiation complex proteins, and proteins involved in the EMT, apoptosis, invasion, and migration. Future research should focus on utilizing these combination therapies using an in vivo model. Overall, this compelling in vitro data suggests that the combination treatment of cisplatin with natural Hsp90 hetero chaperone complex inhibitors like WGA-TA may lower cisplatin doses to reduce potential toxicity. Together, these effects could result in an improved, safer therapy with more durable efficacy for individuals with advanced HNSCC; however, further in vivo translational validation is warranted to better evaluate the clinical potential of such a combination.

## Figures and Tables

**Table 1:** (**A**) 1 MDA-1986 and UMSCC-22B (in bold) cells were treated with cisplatin and WGA-TA, and the cell viability was determined by MTS assay after 72 h. The combination index (CI) values were calculated using CompuSyn. CI < 1.0 is synergistic, CI = 1 is additive, and CI > 1.0 is antagonist. (**B**) 1 MDA-1986 and UMSCC-22B (in bold) cells were treated with cisplatin and WGA-TA and the cell viability was determined by MTS assay after 24 h. The combination index (CI) values were calculated using CompuSyn. CI < 1.0 is synergistic, CI = 1.0 is additive, and CI > 1.0 is antagonist.

**A**				
**Cisplatin** **Dose (μM)**	**WGA-TA** **Dose (μM)**	**Combination** **Effect**	**CI Value**	**Cell Lines**
**1.25**	**2.50**	**0.017**	**0.04**	**UMSCC-22B**
**1.25**	**1.25**	**0.078**	**0.07**	
**1.25**	**0.63**	**0.178**	**0.07**	
**1.25**	**0.31**	**0.473**	**0.12**	
**1.25**	**0.16**	**0.864**	**0.31**	
**1.25**	**0.08**	**0.972**	**0.69**	
**2.50**	**2.50**	**0.017**	**0.04**	
**2.50**	**1.25**	**0.064**	**0.06**	
**2.50**	**0.63**	**0.146**	**0.06**	
**2.50**	**0.31**	**0.490**	**0.13**	
**2.50**	**0.16**	**0.795**	**0.21**	
1.25	2.50	0.020	0.42	MDA-1986
1.25	1.25	0.130	0.72	
1.25	0.63	0.140	0.39	
1.25	0.31	0.410	0.44	
1.25	0.16	0.760	0.54	
1.25	0.08	0.920	0.66	
2.50	2.50	0.010	0.30	
2.50	1.25	0.080	0.55	
2.50	0.63	0.140	0.38	
2.50	0.31	0.600	0.36	
2.50	0.16	0.740	0.28	
**B**				
**Cisplatin** **Dose (μM)**	**WGA-TA** **Dose (μM)**	**Combination** **Effect**	**CI Value**	**Cell Lines**
**1.25**	**5.000**	**0.016**	**0.002**	
**1.25**	**2.500**	**0.212**	**0.010**	**UMSCC-22B**
**2.50**	**5.000**	**0.021**	**0.003**	
**2.50**	**2.500**	**0.239**	**0.012**	
**2.50**	**1.250**	**0.479**	**0.014**	
1.25	5.000	0.008	0.556	
1.25	0.625	0.520	0.710	MDA-1986
2.50	5.000	0.003	0.375	
2.50	1.250	0.310	0.953	
2.50	0.313	0.710	0.523	

## References

[1] Ang K.K., Harris J., Wheeler R., Weber R., Rosenthal D.I., Nguyen-Tan P.F., Westra W.H., Chung C.H., Jordan R.C., Lu C. (2010). Human papillomavirus and survival of patients with oropharyngeal cancer. N. Engl. J. Med..

[2] Miller K.D., Siegel R.L., Lin C.C., Mariotto A.B., Kramer J.L., Rowland J.H., Stein K.D., Alteri R., Jemal A. (2016). Cancer treatment and survivorship statistics, 2016. CA Cancer J. Clin..

[3] Parsel S.M., Grandis J.R., Thomas S.M. (2016). Nucleic acid targeting: Towards personalized therapy for head and neck cancer. Oncogene.

[4] Kelland L. (2007). The resurgence of platinum-based cancer chemotherapy. Nat. Rev. Cancer.

[5] Sacco A.G., Cohen E.E. (2015). Current Treatment Options for Recurrent or Metastatic Head and Neck Squamous Cell Carcinoma. J. Clin. Oncol..

[6] Forastiere A.A. (2008). Chemotherapy in the treatment of locally advanced head and neck cancer. J. Surg. Oncol..

[7] Jhaveri K., Ochiana S.O., Dunphy M.P., Gerecitano J.F., Corben A.D., Peter R.I., Janjigian Y.Y., Gomes-DaGama E.M., Koren J., Modi S. (2014). Heat shock protein 90 inhibitors in the treatment of cancer: Current status and future directions. Expert Opin. Investig. Drugs.

[8] Mahalingam D., Swords R., Carew J.S., Nawrocki S.T., Bhalla K., Giles F.J. (2009). Targeting HSP90 for cancer therapy. Br. J. Cancer.

[9] Sidera K., Patsavoudi E. (2014). HSP90 inhibitors: Current development and potential in cancer therapy. Recent Pattents Anticancer Drug Discov..

[10] Cohen S.M., Mukerji R., Samadi A.K., Zhang X., Zhao H., Blagg B.S., Cohen M.S. (2012). Novel C-terminal Hsp90 inhibitor for head and neck squamous cell cancer (HNSCC) with in vivo efficacy and improved toxicity profiles compared with standard agents. Ann. Surg. Oncol..

[11] Donnelly A., Blagg B.S. (2008). Novobiocin and additional inhibitors of the Hsp90 C-terminal nucleotide-binding pocket. Curr. Med. Chem..

[12] Birkeland A.C., Brenner J.C. (2015). Personalizing Medicine in Head and Neck Squamous Cell Carcinoma: The Rationale for Combination Therapies. Med. Res. Arch..

[13] White P.T., Subramanian C., Motiwala H.F., Cohen M.S. (2016). Natural Withanolides in the Treatment of Chronic Diseases. Adv. Exp. Med. Biol..

[14] Zhang H., Motiwala H., Samadi A., Day V., Aube J., Cohen M., Kindscher K., Gollapudi R., Timmermann B. (2012). Minor withanolides of Physalis longifolia: Structure and cytotoxicity. Chem. Pharm. Bull..

[15] Samadi A.K., Tong X., Mukerji R., Zhang H., Timmermann B.N., Cohen M.S. (2010). Withaferin A, a cytotoxic steroid from Vassobia breviflora, induces apoptosis in human head and neck squamous cell carcinoma. J. Nat. Prod..

[16] Livingstone M., Sikstrom K., Robert P.A., Uze G., Larsson O., Pellegrini S. (2015). Assessment of mTOR-Dependent Translational Regulation of Interferon Stimulated Genes. PLoS ONE.

[17] Chou T.C., Talalay P. (1984). Quantitative analysis of dose-effect relationships: The combined effects of multiple drugs or enzyme inhibitors. Adv. Enzyme Regul..

[18] Pearl L.H., Prodromou C., Workman P. (2008). The Hsp90 molecular chaperone: An open and shut case for treatment. Biochem. J..

[19] Grzmil M., Hemmings B.A. (2012). Translation regulation as a therapeutic target in cancer. Cancer Res..

[20] Furic L., Rong L., Larsson O., Koumakpayi I.H., Yoshida K., Brueschke A., Petroulakis E., Robichaud N., Pollak M., Gaboury L.A. (2010). eIF4E phosphorylation promotes tumorigenesis and is associated with prostate cancer progression. Proc. Natl. Acad. Sci. USA.

[21] Liu T., Li R., Zhao H., Deng J., Long Y., Shuai M.T., Li Q., Gu H., Chen Y.Q., Leng A.M. (2016). eIF4E promotes tumorigenesis and modulates chemosensitivity to cisplatin in esophageal squamous cell carcinoma. Oncotarget.

[22] Topisirovic I., Guzman M.L., McConnell M.J., Licht J.D., Culjkovic B., Neering S.J., Jordan C.T., Borden K.L. (2003). Aberrant eukaryotic translation initiation factor 4E-dependent mRNA transport impedes hematopoietic differentiation and contributes to leukemogenesis. Mol. Cell Biol..

[23] Culjkovic-Kraljacic B., Baguet A., Volpon L., Amri A., Borden K.L. (2012). The oncogene eIF4E reprograms the nuclear pore complex to promote mRNA export and oncogenic transformation. Cell Rep..

[24] Comtesse N., Keller A., Diesinger I., Bauer C., Kayser K., Huwer H., Lenhof H.P., Meese E. (2007). Frequent overexpression of the genes FXR1, CLAPM1 and EIF4G located on amplicon 3q26-27 in squamous cell carcinoma of the lung. Int. J. Cancer.

[25] Silvera D., Arju R., Darvishian F., Levine P.H., Zolfaghari L., Goldberg J., Hochman T., Formenti S.C., Schneider R.J. (2009). Essential role for eIF4GI overexpression in the pathogenesis of inflammatory breast cancer. Nat. Cell Biol..

[26] El-Naggar A.M., Sorensen P.H. (2018). Translational control of aberrant stress responses as a hallmark of cancer. J. Pathol..

[27] Truitt M.L., Ruggero D. (2016). New frontiers in translational control of the cancer genome. Nat. Rev. Cancer.

[28] Yang J., Wang J., Chen K., Guo G., Xi R., Rothman P.B., Whitten D., Zhang L., Huang S., Chen J.L. (2013). eIF4B phosphorylation by pim kinases plays a critical role in cellular transformation by Abl oncogenes. Cancer Res..

[29] Dauphin M., Barbe C., Lemaire S., Nawrocki-Raby B., Lagonotte E., Delepine G., Birembaut P., Gilles C., Polette M. (2013). Vimentin expression predicts the occurrence of metastases in non small cell lung carcinomas. Lung Cancer.

[30] Oliver F.J., de la Rubia G., Rolli V., Ruiz-Ruiz M.C., de Murcia G., Murcia J.M. (1998). Importance of poly(ADP-ribose) polymerase and its cleavage in apoptosis. Lesson from an uncleavable mutant. J. Biol. Chem..

